# The multiple self and psychological openness

**DOI:** 10.3389/fpsyg.2024.1441953

**Published:** 2025-02-12

**Authors:** Hubert Suszek, Maciej Kopera, Andrzej Jakubczyk

**Affiliations:** ^1^Faculty of Psychology, University of Warsaw, Warsaw, Poland; ^2^Department of Psychiatry, Medical University of Warsaw, Warsaw, Poland

**Keywords:** multiple self, unitary self, self-concept, psychological openness, openness to experience, open-mindedness

## Abstract

**Introduction:**

This research identifies and explores two distinct modes of self-experience and their influence on psychological openness. We distinguish between the unitary self-mode, where individuals perceive themselves as cohesive, stable entities, and the multiple self-mode, where they recognize their diverse, context-dependent aspects. These modes represent fundamentally different ways of experiencing and organizing self-knowledge that can be situationally activated. While both modes of self-experience have been theoretically described, their influence on psychological functioning remains empirically unexplored.

**Methods:**

Through five experiments (*N* = 989), we tested whether activation of the multiple self-mode increases psychological openness compared to activation of the unitary self-mode using different experimental manipulations and measures.

**Results:**

Induction of the multiple self enhanced psychological openness compared to induction of the unitary self. This effect was consistently observed across various domains of openness: openness as a state (Study 1, *N* = 204), openness to change (Studies 3 and 4, *N* = 230 and *N* = 184), range of values (Studies 2 and 3, *N* = 212 and *N* = 230), psychological mindedness and decentering (Study 5, *N* = 159). Results consistently showed moderate effect sizes (d = 0.31–0.44) across different operationalizations of both the multiple self-induction and openness measures.

**Discussion:**

These findings indicate that the way in which individuals organize their self-knowledge has important implications for their cognitive and experiential flexibility, contributing to our understanding of personality plasticity and development.

## Introduction

Researchers have long demonstrated sustained interest in multiple representations of the self and their implications. James (1890), for example, distinguished the material, social, and spiritual selves. Some theories center on different universal aspects of the self, while other theories suggest the existence of self-components that are specific to the individual (e.g., Andersen and Chen, 2002; Cantor and Kihlstrom, 1987; Fenigstein et al., 1975; Higgins, 1987; Markus, 1977; Markus and Nurius, 1986; McConnell, 2011; Ogilvie, 1987; Tajfel and Turner, 1979; Triandis, 1989). These theories emphasize the multiplicity of the self, proposing that individuals possess multiple, context-dependent self-aspects that emerge in response to varying social roles, relationships, and environments.

At the same time, the idea of the self as integrated and unified was central to early personality theorists, such as Allport (1961), Maslow (1968), and Rogers (1961), and also featured in developmental frameworks (e.g., Erikson, 1980; Loevinger, 1966). These scholars viewed the self as a core, cohesive structure within the personality that remains stable and independent of the various changing contexts in an individual's life. Additionally, several theories highlight a universal and fundamental need for consistency, emphasizing the effort individuals make to preserve the integrity and unity of the self (Epstein, 1981; Festinger, 1957; Heider, 1958; Kelly, 1955; Lecky, 1945; Swann et al., 1987).

The idea of a central, consistent self appears to persist in the common belief in the unity and coherence of personality. Expressions such as “my real self,” “to be authentic,” or “be yourself” seem to reflect this underlying belief. However, there seem to be situations in which a person is able to perceive two or more of his/her aspects simultaneously. For example, he/she may observe that her confused feelings about something or conflicted desires are related to two visions of herself. He/she may also be surprised by his/her behavior that is indicative of some aspect of his/herself that does not fit with another aspect of his/herself. He/she may also be trying hard to hide or “fight” an unaccepted aspect of his/herself. *Such experiences, where individuals become aware of their inner diversity—recognizing internal conflicts, ambivalence, or engaging in inner dialogues between different aspects of themselves—are well-documented in psychological research on self-concept and internal dialogues* (Hermans and Kempen, 1993; McConnell, 2011).

This apparent tension between theories of self-multiplicity and self-unity, combined with the complex nature of self-experience, poses a significant theoretical challenge in understanding the self-concept. On one hand, individuals often strive for coherence and consistency, yet they are equally capable of recognizing their inner diversity—becoming aware of internal conflicts, ambivalence, or the dialogue between different aspects of the self. These observations suggest that individuals can simultaneously or alternately experience both a unified self and a multiple self, depending on the context. This raises important questions about how these modes of self-experience coexist and interact, and how individuals navigate between stability and flexibility in their self-concept.

We propose that people can function in two distinct modes of self-processing—the unitary self-mode and the multiple self-mode—which can be situationally activated.

In the first mode an individual perceives himself and experiences himself as a unified, integrated, singular, coherent or stable being. In the other, one perceives or experiences one's multiplicity of selves. Due to limited conscious resources, only a small part of self-knowledge is accessible at any given point in time, with one aspect (often the chronically accessible one) dominating resources, as shown on the working self (Markus and Kunda, 1986).

Even though the multiplicity of selves has received considerable attention in social cognition and psychotherapy, no attempts have yet been made to explore unity and multiplicity as two distinct modes of processing information about the self. Sakellaropoulo and Baldwin (2006) suggested that future studies might be considered to empirically examine fluidity among selves, for example, priming multiple selves simultaneously and observing the consequences. *These two modes of processing information about the self are likely to* manifest in an alternating manner and, most importantly, can influence cognition, motivation, and emotion.

We define the unitary self as a cohesive and stable self-concept in which individuals perceive and experience themselves as having core, unchanging characteristics that remain consistent across different contexts and situations. In contrast, we define the multiple self as a view and experience of the self in which individuals recognize and engage with multiple, distinct aspects of their personality. These self-aspects may vary depending on the context, social role, or internal state, allowing for a more flexible and dynamic self-concept.

The unitary self seems to function as the default mode of self-processing, with the multiple self being activated less frequently. It is very common to think about oneself in term of one's unique traits. This tendency is reinforced by a universal and fundamental need for consistency, which motivates people to maintain the integrity and unity of their self-concept (Epstein, 1981). Additionally, individuals often assume their self-unity without question, focusing primarily on maintaining a coherent narrative about who they are (McAdams, 1996).

In contrast, recognizing that multiple self-schemas operate at different times, in different contexts, or even simultaneously requires greater cognitive effort. For example, Hermans and Kempen (1993) argue that accessing the multiplicity of the self often involves active engagement in internal dialogues, which is less automatic than maintaining a unitary perspective. Similarly, the motivation to confirm a unified and consistent self-concept (Swann et al., 1987) may limit individuals' awareness of their inner diversity. Therefore, the multiple self-mode is likely to be less accessible than the unitary self-due to the psychological effort required and the inherent preference for self-coherence.

It is important to clarify that while we discuss the unitary self and multiple self as distinct modes, we do not view them as rigid, mutually exclusive categories. Rather, we conceptualize them as ends of a spectrum of self-experience. Our approach is further informed by research on individual differences in self-pluralism (McReynolds et al., 2000), self-complexity (Linville, 1985), and self-concept differentiation (Donahue et al., 1993). These constructs highlight the varying degrees to which individuals experience and organize multiple self-aspects. Self-pluralism refers to the extent to which individuals perceive themselves as having multiple selves that vary across situations. Self-complexity describes the number and distinctiveness of self-aspects in an individual's self-concept. Self-concept differentiation refers to the degree to which individuals see themselves as having different personality characteristics across various social roles or situations. These individual differences suggest that people may have predispositions toward experiencing themselves as more unitary or more multiple. However, we propose that regardless of these trait-like tendencies, individuals can shift along the unitary-multiple spectrum depending on context and current psychological state.

At one end of the spectrum, the unitary self-mode represents a state where individuals perceive themselves as having a highly integrated, consistent sense of self across contexts. At the other end, the multiple self-mode represents a state where individuals are acutely aware of their different, potentially contradictory self-aspects. Most experiences of self-likely fall somewhere between these extremes. For instance, an individual high in self-complexity might generally be aware of their multiple self-aspects but experience moments of greater self-unity in certain contexts. Conversely, someone low in self-pluralism might occasionally become more aware of their different selves in situations that highlight various social roles.

The conceptualization of unitary self and multiple self as ends of a spectrum suggests that these modes could potentially be primed or temporarily activated. This theoretical possibility aligns with established research showing consequences of activation or priming of different selves, e.g., self-schemas (Markus, 1977), ideal selves (Higgins, 1987), relational selves (Andersen and Chen, 2002), or independent-interdependent self-construals (e.g., Oyserman and Lee, 2008). In a similar vein, it may be possible to temporarily shift individuals' self-perception toward either a more unitary or more multiple experience of self. Such an approach could allow for the examination of cognitive and behavioral correlates associated with these different modes of self-experience.

The distinction between unitary and multiple self-modes raises the question of when and how individuals switch between these modes. While this is an intriguing area for further exploration, understanding the specific conditions that trigger such transitions is beyond the scope of the present study. Prior research on self-concept variability provides valuable insights into how individuals shift their self-perception in response to various situational and emotional factors. Studies have demonstrated that such shifts can be influenced by contextual cues, emotional states, interpersonal dynamics, and environmental demands (Markus and Wurf, 1987; McConnell, 2011; Roberts and Donahue, 1994). We propose that a similar dynamic may govern the transition between multiple self-mode and unitary self-mode. It is plausible that the multiple self-mode is activated most often in contexts involving internal conflict, feedback from others about one's personality, or intensive self-reflection, as these situations encourage individuals to explore and reconcile different aspects of their self-concept. Particularly, the process of deep self-reflection, as observed in psychotherapy, may play a critical role in activating the multiple self-mode.

## Multiplicity of selves in psychotherapy

Many psychotherapy approaches refer to the idea of multiplicity of selves and try to understand psychopathological processes in terms of inner multiplicity. Psychotherapists proposed the existence of both universal and idiographic distinctions among self-aspects. In psychotherapeutic work the inner multiplicity of the patient is brought to light and relations between different subsystems are examined. Among such approaches one should enlist gestalt therapy (Perls, 1973), voice dialogue (Stone and Winkelman, 1985), ego states therapy (Watkins and Watkins, 1997), internal family systems therapy (Schwartz, 1995), schema therapy (Young, 2003), and various schools that refer to the so-called subpersonalities (e.g., Rowan, 1990). Different names have been used for each subsystem in these approaches. Some examples are: “parts,” “subselves,” “ego states,” “voices,” “roles,” “alter egos,” “potentials,” “others,” “personas,” “mind states,” and “schemata.”

It can be assumed that during many therapies in different approaches therapists not only apply techniques aimed to support some therapeutic processes (e.g., helping a client to get to know his unknown aspect) but at the same time they stimulate or even teach clients a specific way of thinking about oneself. Clients learn how to understand or symbolize their experience in terms of the multiplicity of selves, effectively activating the multiple-self mode.

Investigating the consequences of this mode of self-organization holds significant theoretical and practical importance. By isolating the multiple-self mode from other therapeutic processes and contextual factors, researchers can systematically examine its specific effects within a controlled experimental setting. This approach allows us to test whether activating the multiple-self mode produces outcomes aligned with therapeutic goals, such as fostering greater openness to experience and psychological flexibility.

Openness to experience, characterized by cognitive and experiential flexibility, is central to psychological wellbeing and personal growth. Clinically, openness has been linked to greater adaptability, increased self-awareness, and a heightened tolerance for ambiguity—all of which are critical for effective therapy and personal development (McCrae and Costa, 1997). For example, clients with higher openness are more likely to engage in self-reflection and benefit from exercises promoting cognitive flexibility and experiential learning. In therapeutic contexts, openness supports processes such as self-exploration, emotional processing, and self-acceptance (Hayes et al., 2011; Rogers, 1961). Similarly, openness fosters resilience and adaptability, enabling individuals to navigate internal conflicts and reconcile diverse self-aspects—a process central to the multiple-self mode.

Openness is conceptually opposed to behavioral rigidity—a hallmark of many personality disorders. Behavioral rigidity reflects a fixed and inflexible self-concept, limiting an individual's ability to adapt to changing circumstances or integrate new perspectives. In contrast, activating the multiple-self mode promotes the exploration and integration of diverse self-aspects, fostering a more flexible and adaptive approach to self-concept. Psychological flexibility is widely recognized as a fundamental aspect of mental health and has been empirically linked to enhanced wellbeing and reduced stress (Wersebe et al., 2018). Flexible individuals are better equipped to life's shifting demands, reconfigure mental resources, shift perspectives, and balance competing desires and need (Kashdan and Rottenberg, 2010). Understanding how the multiple-self mode facilitates flexibility and openness offers valuable insights into the mechanisms underlying therapeutic change, addressing both theoretical questions and practical applications in clinical settings. In the next section we will outline the possibility of changing openness to experience.

## Openness to experience as a changeable factor

Openness to experience is a dimension of personality, belonging to the five-factor model of personality and is usually portrayed as an intrapsychic dimension, describing individual differences in the structure and functioning of the mind (McCrae and Costa, 1997). Openness is manifested in “the breadth, depth, and permeability of consciousness, and in the recurrent need to enlarge and examine experience” (McCrae and Costa, 1997, p. 826). Openness is a wide-ranging and general trait, characterized by vivid fantasy, artistic receptiveness, emotional depth, versatility in behavior, curiosity of the intellect, and non-traditional viewpoints. Openness to experience has important implications for psychotherapy (Miller, 1991). Open individuals have less rigid views of right and wrong or of appropriate and inappropriate behaviors (Black, 1990). Openness to experience has been associated with positive therapeutic outcomes in various psychotherapeutic modes. In a meta-analysis, patients' openness (vs. defensiveness) in therapy was related to positive outcomes (Bergin and Garfield, 1994).

The last decades of research on personality development have been dominated by a controversy on the general changeability of the Big Five traits (Srivastava et al., 2003). McCrae and Costa (1997) argued that personality traits are biologically programmed entities that cannot be altered. In contrast, some research has demonstrated that personality traits are malleable and change in response to a variety of contextual and environmental factors, including life experiences (Roberts and Mroczek, 2008) and that people are able to volitionally change their personality traits (Hudson and Fraley, 2015). In some such studies the plasticity of openness to experience was addressed.

It has been demonstrated that openness to experience declines after marriage (Specht et al., 2011), after unemployment (Boyce et al., 2015), after chronic disease (Jokela et al., 2014), and increases after upward job changes into professional and managerial positions (Nieß and Zacher, 2015), after 1 or 2 semesters sojourning among university students (Zimmermann and Neyer, 2013), and after mystical experiences induced by administration of psylocybin with a 1 year follow-up (MacLean et al., 2011).

A number of intervention studies have suggested that personality, especially openness to experience is amenable to change. It has been demonstrated that openness to experience increased after a reasoning training program (Jackson et al., 2012), a training course in volunteering (Mühlig-Versen et al., 2012), psychotherapy (Piedmont, 2001), coaching sessions (Martin et al., 2014), and even after a fiction reading assignment (Djikic et al., 2009).

The above-mentioned research was conducted in accordance with the classic trait-based approach to personality and openness was measured with validated questionnaires constructed for measuring stable traits. From the perspective of this approach one can expect that attempts to activate the multiple self may target openness change at the facet, rather than trait level. Such activations may lead to changes in openness-related behaviors.

However, in the light of social-cognitive approaches, openness is a conditional disposition which can manifest when certain conditions arise (Mischel and Shoda, 1995). Several studies have found that individuals alter their personality as they move from one social context to another (e.g., from family to friends to work colleagues; Donahue and Harary, 1998; Robinson, 2009). For example, Robinson (2009) found that participants rated their personality as less open with parents than with work colleagues or friends. From this perspective experimental manipulations of self-concept may be sufficient for creating such conditions. Another difference between the classic trait approach is that the social-cognitive approach goes beyond broad personality traits and includes such factors as goals, beliefs, values or attitudes, which are more amenable to change.

A similar perspective is offered by the whole trait theory (Fleeson, 2001). According to this theory, the descriptive aspects of traits result from the accumulation of trait expressions in daily life, referred to as a “personality state.” A personality state is characterized by possessing identical affective, cognitive, and behavioral elements as a related trait (Zillig et al., 2002) but operates over a briefer period. These states can be assessed akin to personality traits, employing comparable content and scales, yet focusing on describing the individual at a specific moment rather than in a broader context.

## The multiple self and psychological openness

In exploring links between self-organization and openness, it is important to distinguish between openness to experience as traditionally defined in personality psychology as one of the Big Five traits, and psychological openness as a broader phenomenon encompassing various manifestations of cognitive and experiential flexibility (Hayes et al., 2003; Kashdan and Rottenberg, 2010). In this study, we use the broad concept of psychological openness, which encompasses diverse forms of openness such as openness to new values, openness to change, psychological reflexivity, decentering, and acceptance. Here, psychological openness describes a range of states and attitudes associated with cognitive and emotional flexibility. This broader conceptualization allows us to examine how self-organization influences different forms of openness beyond trait-level tendencies.

We hypothesize that activation of the multiple self-increases openness states through several mechanisms:

Firstly, recognizing multiple aspects of oneself likely enhances cognitive flexibility, as individuals become adept at switching between different self-views and perspectives. This flexibility closely aligns with the adaptability characteristic of openness to experience.

Secondly, holding potentially conflicting self-views may foster a greater tolerance for ambiguity, a hallmark of openness. As individuals reconcile diverse aspects of their identity, they may become more comfortable with complexity and contradictions in their self-concept and with uncertainty, which can enhance their overall curiosity and willingness to explore unfamiliar situations.

Thirdly, an expanded self-concept resulting from acknowledging multiple self-aspects may lead to broader interests and a willingness to engage with diverse experiences, directly contributing to openness.

Lastly, the process of reflecting on multiple selves likely promotes enhanced self-reflection and curiosity about one's inner world, key components of openness to experience.

These processes align closely with key aspects of openness to experience, such as intellectual curiosity, preference for novelty, and willingness to engage with diverse ideas and experiences (McCrae and Costa, 1997). By encouraging individuals to recognize and engage with multiple facets of their identity, we posit that a multiple self-perspective creates a cognitive and emotional state more conducive to openness.

One may ask whether there is support for our hypothesis in results from studies on individual differences in the structure of self-concept. Two variables of this kind, widely debated within the field of psychology, are self-complexity and self-concept differentiation. The first reflects the number of distinct self-aspects within self-concept (Linville, 1985) while the second refers to an individual's inclination to see themselves as having varied personality traits in different social roles (Donahue et al., 1993). Donahue et al. (1993) found no correlation between self-concept differentiation and openness to experience. No studies have reported a direct connection between self-complexity and openness. However, serious methodological concerns have been voiced in relation to the measurement of those variables (see, for review, Pilarska and Suchańska, 2015).

Indirect support for our general expectation is provided by studies on internal dialogues. Puchalska-Wasyl et al. (2008) conducted studies on individual differences in the internal dialogical activity which represents the intensity and richness of dialogs between various selves with imaginary interlocutors. The results indicated that individuals who engaged in inner dialogues exhibited a significantly higher level of openness, as measured by the NEO PI-R, compared to those who did not engage in inner dialogues. The method of measurement of internal dialogical activity included a list of potential selves and imaginary interlocutors and we can assume that they represent various selves, especially relational selves. In a second study where another measure of the internal dialogical activity was used, a moderate positive correlation (0.44) was found between the intensity of such activity and openness. Another study conducted by these authors showed that internal dialogical activity fulfilled several positive functions like self-improvement, insight or self-guidance.

There are also experimental studies which indirectly speak for the connection between induced multiplicity of selves and openness. Staudinger and Baltes (1996) observed in an experimental study that conducting an inner dialogue about a difficult life problem resulted in significantly higher levels of wisdom-related performance than just thinking about the problem. Oleś et al. (2010) conducted experiments on temporal dialogues. In several studies authors activated two or three selves (past self-vs. present self-vs. future self) by confronting and switching between them. They observed that this manipulation increased the state of curiosity, increased the meaning of life and decreased anxiety, depression and anger. The researchers deduced that by engaging with their past, future, and present selves, individuals can clarify their goals, values, and desires, thereby impacting their sense of life's meaning. Although those studies dealt with relations between self-aspects, we think that the mode of self-multiplicity was directly or indirectly activated.

It is important to note that while both self-organization (including self-complexity, self-pluralism, and self-concept differentiation) and psychological openness have been extensively studied separately, research examining their direct relationships is surprisingly lacking (with Donahue et al., 1993 being one exception, finding no correlation between self-concept differentiation and openness to experience). Therefore, our research provides the first experimental examination of how inducing different modes of self-experience influences psychological openness, moving beyond correlational designs to test causal relationships. This experimental approach allows us to examine whether activating different modes of self-experience can influence various forms of openness.

In summary, the multiple self-construct fosters openness by promoting cognitive flexibility, self-exploration, and dialogue between diverse self-aspects, leading to increased curiosity and a willingness to engage with novelty. This study aims to empirically test this theoretical link, potentially unveiling a novel pathway to enhancing psychological openness.

## Purpose of the study

The main purpose of the study was to examine whether activation of the multiple self-increases psychological openness compared to activation of the unitary self. We selected six dimensions that represent distinct but complementary manifestations of psychological openness. State openness captures immediate flexibility in experiencing and processing information. Openness to values reflects receptivity to different axiological perspectives. Openness to change represents behavioral flexibility and adaptability. Psychological reflexivity captures the cognitive component of openness—the ability to examine one's thoughts and experiences. Decentering represents the meta-cognitive aspect of openness—the ability to step back and observe experiences from different perspectives. Finally, acceptance reflects the emotional component of openness—the ability to embrace diverse experiences without judgment. Together, these dimensions allow us to examine how multiple self-activation might influence psychological openness across cognitive, emotional, and behavioral domains. We expected that perceiving or experiencing one's multiple self (as opposed to unitary self) increases various aspects of psychological openness, namely: (a) openness as a state; (b) openness to values; (c) openness to change; (d) psychological reflexivity; (e) decentering; and (f) acceptance.

## Study 1

In the first study, our aim was to test whether activation of the multiple self-increases openness to experience as a state compared to activation of the unitary self. We induced the multiple self or the unitary self and then measured the reported intensity of openness to experience. We expected that induction of the multiple self would cause individuals to describe themselves as more open to experience compared to individuals in whom the unitary self was induced. This hypothesis was grounded in several theoretical considerations. First, while openness to experience is typically conceived as a stable personality trait, research shows it can also manifest as a temporary psychological state influenced by situational factors (Fleeson, 2001). Second, the multiple self-mode inherently involves recognizing and integrating diverse aspects of the self, which requires switching between different self-views and perspectives. This cognitive and experiential flexibility aligns with the conceptualization of state openness as a dynamic, momentary willingness to engage with novelty and complexity. Finally, the cognitive effort involved in recognizing diverse self-aspects may broaden attention to novel experiences and perspectives, increasing openness at a state level.

In addition, we used a mood measure after the manipulation to rule out the possibility that it was a mediator of the expected effect. We based this on the assumption that some individuals experiencing conflicts around self-image might feel a sense of relief resulting from focusing on their multiple sides, which might be responsible for an increase in mood. On the other hand, one might think that just focusing on one's many sides might trigger conflict-related experiences such as anxiety and thus lower mood. To rule out these two possibilities we decided to assess mood after the experimental manipulation.

### Method

#### Participants

A total of 204 students (82% female) aged 19 to 57 (*M* = 30.7 years, *SD* = 12.8) from the University of Warsaw participated in the study in exchange for small gifts.

#### Measures

We measured openness as a state using the 12-item Openness to Experience subscale derived from the Polish version of the NEO-FFI questionnaire (Costa and McCrae, 1992; Zawadzki et al., 1998). This subscale is one of five subscales representing the five dimensions of personality that make up the 60-item questionnaire. Every question is assessed using a 5-point scale where 1 corresponds to “strongly disagree,” and 5 corresponds to “strongly agree.”

#### Procedure

Subjects were invited to the laboratory and were then randomly assigned to either the multiple self-condition or the unitary self-condition. In the multiple self-condition, we asked participants to isolate and then describe the different sides of their personality using a few sentences. In the unitary self-condition, on the other hand, we asked subjects to freely describe their most characteristic properties that are invariant.

After the manipulation, subjects described their current mood using a 1-item 7-point scale (1 = negative, 7 = positive).

We then asked subjects to complete the openness to experience subscale of the NEO-FFI questionnaire.

Finally, we checked whether participants guessed the purpose of the study and whether they were aware that an experimental manipulation had occurred. For this purpose, they answered two questions (Bargh and Chartrand, 2000): (a) what they thought the purpose of the study was and what the hypothesis was, and (b) whether the tasks they performed were related, and if so, how. After the study, participants were given an explanation of what the study was about.

### Results and discussion

None of the subjects guessed the purpose of the study or how the two parts of the study were connected.

To test whether the manipulation was successful, we looked at how people in both conditions described themselves. We excluded six participants who did not seem to understand the instructions and five participants who described two sides of their personality, leaving only participants who characterized at least three aspects of their personality in the experimental condition.

Mood did not change under the manipulation—subjects in the multiple self-condition did not differ in mood (*M* = 4.38; *SD* = 1.4) from subjects in the unitary self-condition (*M* = 4.52; *SD* = 1.34), *t*_(191)_ = 0.74; *p* = 0.46; *d* = 0.11. This result rules out the possibility that mood was a mediator of the expected effect.

We also tested for a statistically significant association between the manipulation and gender distribution, but the chi-square test did not reveal a significant result: χ^2^_(2, N = 193)_ = 1.75, *p* = 0.417.

As predicted, people in whom the multiple self was induced presented a higher degree of openness to experienc*e* (*M* = 44.84; *SD* = 6.05) compared to people in whom the unitary self was induced (*M* = 42.29; *SD* = 5.63), *t*_(191)_ = 2.57; *p* = 0.003, *d* = 0.44. The obtained result shows that thinking about oneself in terms of the multiple self makes the person self-report higher openness to experience. This effect occurred in the case of a measure normally used to capture a stable trait. We think that seeing oneself as someone with different sides causes one to see the richness of one's personality.

## Study 2

The first study showed that induction of the multiple self-increases openness to experience as a state. In the next study, we intended to test whether focusing on the multiple self-increases openness to valuing diversity. Values represent core guiding principles often tied to an individual's sense of self and identity (Schwartz, 1992). We hypothesized that due to induction of the multiple self, individuals should be more open to identifying with a wider range of values. This hypothesis was grounded in several theoretical considerations. First, when individuals recognize different aspects of their personality, they may become more receptive to diverse values that align with these distinct self-aspects. Second, engaging with multiple self-aspects may foster tolerance for complexity in one's value system. Third, acknowledging multiple self-aspects might broaden one's perspective beyond familiar values, making individuals more willing to consider new principles and beliefs.

We decided to use a different method of manipulation of multiple vs. unified self, one in which respondents could choose terms from a larger pool. In this way, we wanted to make the manipulation faster and easier for the respondents.

We also chose to measure the effectiveness of the manipulation more precisely. Instead of checking whether subjects extracted and described the aspects of their self, we intended to ask subjects whether they felt like they were looking at the different sides of their personality during the experimental manipulation.

### Method

#### Participants

A total of 212 University of Warsaw students (8% female) aged 19–49 (*M* = 26.7; *SD* = 7.6) participated in the study and were scored for participation.

#### Measures

To measure the range of values, we used Scheller's Value List (Brzozowski, 1995). It consists of 50 values. In the present study, we asked the respondents to select from these values the ones that are important to them.

#### Procedure

Participants were invited to the laboratory and were then randomly assigned to a condition in which the multiple self was elicited or a condition in which the unitary self was elicited. The manipulation was based on a modified version of the Linville (1985) and Trzebińska (2002) sorting task. In the first step in both conditions, participants selected from a list of terms that fit their personality. They then arranged these terms in an appropriate manner. In the multiple self-condition, they arranged these terms into groups corresponding to their personality aspects. In the unitary self-condition, they created a ranking of these terms—arranging them from most important to less important. This two-step procedure was designed to maintain a similar number of selected terms in both conditions.

As in the previous study, after the manipulation, subjects rated their current mood on a 1-item, 7-point scale (1 = negative, 7 = positive).

In the next step, we tested the effectiveness of the manipulation. For this purpose, subjects answered two 1-item questions (“To what extent did you focus on different sides of your personality during that task?” and “To what extent did that task allow you to look at different sides of your personality?”) along with 7-point scales (1 = not at all; 7 = very much). Responses to these two questions were combined to produce a single indicator.

In the next step, participants selected from a list of 50 values those values that are important to them.

Finally, we checked whether participants guessed the purpose of the study and whether they were aware of the manipulation (Bargh and Chartrand, 2000). After the study, participants were given explanations about the true purpose of the study.

### Results and discussion

None of the participants guessed the purpose of the study or its experimental nature. Four subjects did not perform the experimental manipulation task as instructed and were excluded. In the multiple self-condition, all subjects (except those excluded) described at least three sides of their personality, which leads to the conclusion that the manipulation was effective.

The experimental manipulation proved to be effective. Subjects in the multiple self-condition claimed that the task allowed them to focus on and look at different sides of their personality to a greater extent (*M* = 5.24; *SD* = 1.08) than subjects in the unitary self-condition (*M* = 4.86; *SD* = 1.35), *t*_(210)_ = 2.26; *p* = 0.025; *d* = 0.31.

Mood did not change under the manipulation—subjects in the multiple self-condition did not differ in mood (*M* = 4.41; *SD* = 1.43) from subjects in the unitary self-condition (*M* = 4.53; *SD* = 1.24), *t*_(210)_ = 0.66; *p* = 0.51; *d* = 0.09. This result rules out the possibility that mood was a mediator of the expected effect.

As expected, activation of the multiple self-increased the number of values chosen as one's own (*M* = 22.35; *SD* = 7.91) compared to the condition in which the unitary self was induced (*M* = 19.14; *SD* = 7.5), *t*_(210)_ = 3.02; *p* = 0.003; *d* = 0.41. This suggests that induction of the multiple self-causes subjects to consider more values as important. We think that in doing so, individuals expand their openness to different areas of life, change their attitudes toward people and things to more positive ones, and likely see more life goals as worth pursuing.

## Study 3

Study two showed that individuals in whom the multiple self was elicited considered a greater number of values to be important. In the next study, we intended to examine whether activation of the multiple self-affected values in terms of content, specifically, whether it affected values related to openness. We expected that as a result of multiple self-induction, individuals should identify more values associated with openness than in the unitary self-condition. This hypothesis was based on several theoretical considerations. First, the dialogic nature of the multiple self-mode might specifically support openness to values that involve change and transcendence of self-interest. Second, recognizing different self-aspects may facilitate tolerance for contradictions in one's value system, making individuals more open to values that challenge their current perspectives. Third, engaging with multiple self-aspects might help individuals appreciate the complexity of their value system, reducing the rigidity of existing value hierarchies and making them more receptive to values associated with openness and growth.

We also decided to make several changes to the procedure. First, we used a more structured manipulation of the multiple self. The two manipulations used in previous studies did not include instructions that specified how many aspects of the self the subjects were to focus on. In the present study, the manipulation was modified so that when the multiple self was elicited, subjects actually described several aspects of the self.

Second, we chose to present the experimental manipulation to subjects in a “positive” way. This applies to both conditions. We cannot rule out the possibility that encouraging some individuals to focus on their inner diversity might induce anxiety, ambiguity, or feelings of confusion about who one is. To prevent these potential feelings from becoming a confounding variable, both modes of thinking about oneself were presented to the subjects as natural.

### Method

#### Participants

A total of 230 participants (60% female) aged 17–73 years (*M* = 31.91; *SD* = 15.27), of whom 51% had a university education, 47% had a high school education, and 2% had a primary school education, participated in the online survey. Selection was random; the survey link was distributed using a snowball method. Respondents did not receive gratuities for participation.

#### Measures

To measure openness to change, we used the 21-item version of the Portrait Values Questionnaire (PVQ; Schwartz, 2003). It consists of 10 value types: security, power, achievement, hedonism, stimulation, self-direction, universalism, benevolence, tradition, and adaptation. The questionnaire allows us to analyze the value system in terms of four meta-categories, constituted by two dimensions (Schwartz and Boehnke, 2004): openness to change vs. conservativeness, strengthening the self-vs. transcending the self. The meta-category of openness to change was the object of our interest.

#### Procedure

The study was conducted using the Qualtrics survey research platform. Subjects were randomly assigned to either the multiple self-condition or the unitary self-condition. For the manipulation, we used a modified version of the twenty-statement test (Kuhn and McPartland, 1954). This test normally serves as a measure of individual differences in the concept of self. Subjects are asked to describe themselves in terms of 20 statements. In our study, this test served as an experimental manipulation. In the unitary self-condition, participants completed a classic version of the test, expanded to 21 statements. In the multiple self-condition, participants described seven predetermined parts of their personality. Each part was described using three statements, making a total of 21 statements. The seven designated parts of personality were: your inner self, you to strangers, you at work/university, you in a social/group setting, you in a close relationship, you as a friend, and your dark/disliked side. We selected these seven designated personality parts based on a previous pilot study (*N* = 122) in which participants described themselves using Linville (1985) sorting task. The most commonly formed personality sides were selected as universal and used to create the experimental manipulation.

The manipulation in both conditions was presented to the subjects in a “positive” manner, i.e., the instruction in the multiple self-condition included the information that “it is natural for us to have many different personality sides, aspects, or different facets,” whereas in the unitary self-condition it included the information that “it is natural for us to have some unchanging characteristics that distinguish us from other people.”

As in the previous study, after the manipulation, subjects rated their current mood on a 1-item, 7-point scale and then answered the same questions designed to test the effectiveness of the manipulation. Finally, we checked whether participants guessed the purpose of the study and whether they were aware of the manipulation using the same questions as in the previous study (Bargh and Chartrand, 2000).

### Results and discussion

None of the subjects guessed the purpose of the study or its experimental nature.

The experimental manipulation proved to be effective. Subjects in the multiple self-condition claimed that the task allowed them to focus on and look at different sides of their personality to a greater extent (*M* = 5.07; *SD* = 1.16) than subjects in the unitary self-condition (*M* = 4.21; *SD* = 1.17), *t*_(228)_ = 5.57; *p* < 0.001; *d* = 0.74.

As in the previous studies, mood did not change under manipulation—subjects in the multiple self-condition did not differ in mood (*M* = 4.73; *SD* = 1.3) from subjects in the unitary self-condition: (*M* = 4.51; *SD* = 1.6), *t*_(228)_ = 1.17; *p* = 0.24; *d* = 0.15. This result contradicts the possibility that mood was a mediator of the expected effect.

To calculate the severity of each of the 10 values, we averaged the responses belonging to each value.

Results showed that individuals in whom the multiple self was induced reported higher levels of openness to change (*M* = 3.76; *SD* = 0.88) compared to individuals in whom the unitary self was induced (*M* = 3.54; *SD* = 0.72), *t*_(228)_ = 2.12; *p* = 0.035; *d* = 0.27, and higher levels of transcending self (*M* = 4.70; *SD* = 0.73) compared to individuals in whom the unitary self was elicited (*M* = 4.4; *SD* = 0.81), *t*_(227)_ = 2.91; *p* = 0.004; *d* = 0.39. We did not observe any differences between these conditions in terms of conservativeness (*M* = 3.95; *SD* = 0.94 vs. *M* = 3.89; *SD* = 0.74; *t*_(226)_ = 0.54; *p* = 0.59; *d* = 0.07) and reinforcing self (*M* = 3.71; *SD* = 1.1 vs. *M* = 3.65; *SD* = 1.09; *t*_(227)_ = 0.45; *p* = 0.65; *d* = 0.05).

In addition, we decided to test whether the values measured differently from the previous study also have a different range depending on the condition. For this purpose, we calculated the mean intensity of all values. Subjects in whom the multiple self was elicited reported higher mean value intensity (*M* = 3.99; *SD* = 0.54) compared to subjects in whom the unitary self was elicited (*M* = 3.83; *SD* = 0.45), *t*_(227)_ = 2.36; *p* = 0.012; *d* = 0.32.

As predicted, we found that inducing the multiple self-increased the importance of the meta-category openness to change relative to the condition where the unitary self was induced. That is, in the former condition, subjects declared greater importance of values related to independence of thoughts and actions and readiness to change.

In addition, we observed that subjects in the multiple self-condition showed higher levels of self-transcendence compared to subjects in the unitary self-condition. For those in whom we induced the multiple self, values related to the wellbeing and interests of others were more important.

We also observed that Individuals in whom the multiple self was elicited reported higher mean intensities of all values compared to individuals in whom the unitary self was elicited. Thus, they showed an overall higher openness to values, regardless of their content.

Results show that the induction of the multiple self makes people more prone to change. They seem to free themselves from a fixed vision of themselves for a while. The same applies to self-transcendence. After the induction of the multiple self, people are more prone to transcend the old version of themselves. Lastly, the induction of the multiple self-produced similar results as those obtained in Study 2. Participants identify with a wider range of values.

## Study 4

Study three showed that individuals in whom multiple selves were induced showed higher openness to values. In particular, we found that these individuals were more positive about the possibility of making changes in their lives. In a subsequent study, we intended to test more directly whether activation of the multiple self-influenced openness to change that might occur in the subjects' lives in various areas. We expected that the activation of the multiple self would cause individuals to self-report higher openness about changes in different areas of their lives compared to individuals in whom the unitary self was induced. This hypothesis was grounded in several theoretical considerations. First, the multiple self-mode inherently involves recognizing the dynamic and context-dependent nature of the self, which may reduce attachment to static or rigid identities and facilitate readiness for change. Second, activating multiple self-aspects allows individuals to envision alternative behaviors or lifestyles that align with different self-aspects, potentially increasing openness to personal transformation. Third, engaging with multiple self-aspects challenges rigid self-concepts and promotes a more dynamic self-view, making individuals more open to considering and embracing change in different life domains.

### Method

#### Participants

A total of 184 subjects (87% female) aged 18–64 years (*M* = 24.76; *SD* = 7.95) participated in the online survey, of whom 49% had a university education, 46% had a high school education, and 5% had a primary education. Selection was random; the survey link was distributed using a snowball method. Respondents did not receive gratuities for participation.

#### Measures

We assessed openness to change using a measurement that was designed specifically for this study. Participants rated the extent to which they were open to change in five life areas (daily schedule, occupation, eating habits, hobbies, and political views). They rated each change on a 7-point scale (1 = no openness at all, 7 = total openness). The overall score is the average of the openness to change ratings in each domain. The reliability of the created scale was 0.73.

#### Procedure

The study was conducted using the Qualtrics survey research platform. Subjects were randomly assigned to the multiple self-condition or the unitary self-condition. As an experimental manipulation, we used the same technique as in the previous study. As in the previous studies, after the manipulation, subjects rated their current mood on a 1-item, 7-point scale and then answered the same questions designed to test the effectiveness of the manipulation. Finally, we checked whether participants guessed the purpose of the study and whether they were aware of the manipulation using the same questions as in the previous studies (Bargh and Chartrand, 2000).

### Results and discussion

None of the subjects guessed the purpose of the study or its experimental nature.

The experimental manipulation proved to be effective. Subjects in the multiple self-condition claimed that the task allowed them to focus on and look at different sides of their personality to a greater extent (*M* = 5.11; *SD* = 1.16) than subjects in the unitary self-condition (*M* = 4.62; *SD* = 1.06), *t*_(182)_ = 2.94; *p* = 0.004; *d* = 0.44.

As in the previous studies, mood did not change under manipulation—subjects in the multiple self-condition did not differ in mood (*M* = 4.67; *SD* = 1.27) from subjects in the unitary self-condition (*M* = 4.41; *SD* = 1.29), *t*_(182)_ = 1.4; *p* = 0.16; *d* = 0.2, which rules out mediation.

As predicted, individuals who were induced with the multiple self-reported higher levels of openness to change (*M* = 4.77; *SD* = 1.13) compared to individuals who were induced with the unitary self (*M* = 4.36; *SD* = 1.29), *t*_(182)_ = 2.32; *p* = 0.021; *d* = 1.13. This suggests that induction of the multiple self-causes individuals to report greater openness to change in different areas of their lives. Thus, they are less attached to their current lifestyles. Most likely, their prediction of the future becomes more flexible, and includes more possibilities.

## Study 5

The purpose of the last study was to examine whether induction of the multiple self affects three very broadly defined manifestations of psychological openness. We hypothesized that inducing the multiple self would make individuals more reflective, decentered, and accepting of their experiences compared to individuals in whom the unitary self was induced. This hypothesis was grounded in distinct theoretical considerations for each dimension.

The first of these is psychological mindedness. Open individuals are simultaneously aware of their diverse thoughts, feelings, and impulses (Costa and McCrae, 1992). It is also possible that the same occurs with larger units like self-aspects. Thus, it can be speculated that more open individuals are characterized by higher psychological mindedness. Psychological mindedness is the capacity to recognize connections between thoughts, emotions, and actions, with the aim of understanding the meanings and origins of one's experiences and behavior (Appelbaum, 1973). The activation of the multiple self makes it possible to see relationships between different selves. This can lead to insight about inner conflicts or ambivalence. It also makes it possible to see connections between the various selves, behaviors or experiences in various contexts or in different relationships. One can also recognize connections between different functions within each aspect, like the recognition that one particular aspect may be connected with a belief, value or dominant feeling. Activation of the multiple self may also strengthen the feeling of curiosity of one's inner world. When this mode of processing appears in a psychotherapeutic context it can even make someone feel intrigued by oneself or curious about how the mind works. Understanding more about oneself, seeing more complexity or variety in one's psyche or other minds can make one less rigid and more open to change.

The second manifestation of openness that we intended to test was decentering (similar constructs: *metacognitive awareness, cognitive distancing, cognitive defusion, detached mindfulness, reperceiving, and observing ego*). This is a metacognitive capacity to shift an experiential perspective from within one's subjective experience to that experience (Bernstein et al., 2015). It has been conceptualized as a trait or state. *Activating the multiple self-likely fosters* a detached or observant perspective of one's aspects. It makes it possible to observe oneself with some distance and dis-identification. By noticing one's many versions, it's possible to detach from immediate self-experience, altering its inherent nature. From this meta-perspective, it is easier to recognize many possibilities or potentials of the many selves, in that one can broaden his experience. This can also create a feeling of more choice. From this perspective, it is also possible to recognize new things within the self-concept and it is possible to experience one's self-aspects as more temporary or flexible, less absolute, and unalterable. This process ultimately encourages individuals to perceive oneself as more open.

The third selected manifestation of openness is acceptance. It denotes the ability to experience thoughts, feelings, and physical sensations without making judgments or evaluations and without the need to avoid, change, or control them (Baer, 2003). *Activating the multiple self may enable individuals* to perceive both the positive and the negative or problematic selves. Seeing both one's strengths and weaknesses makes it easier to tolerate the weaknesses. One can think that one is not entirely good or bad but can have good and bad sides of personality. This can lower self-criticism. Especially recognizing both sides of a conflict can make the conflict less disturbing because there is a lesser need to defensively escape from the conflict. Recognizing many selves may also make one's inner life less strange or unknown. Each of these mechanisms can be accounted for as both a trait and a state.

The purpose of the present study was to examine whether induction of the multiple self-increases psychological mindedness, decentering, and acceptance, which we view as manifestations of psychological openness, broadly defined. We expected that inducing the multiple self would make individuals more reflective, decentered, and accepting of their experiences compared to individuals in whom the unitary self was induced.

### Method

#### Participants

A total of 159 subjects (63% female) aged 17–72 years (*M* = 32.08; *SD* = 15.02) participated in the online survey, of whom 51% had a university education, 47% had a high school education, and 2% had a primary education. Selection was random; the survey link was distributed using a snowball method. Respondents did not receive gratuities for participation.

#### Measures

For this study, we constructed an instrument measuring the three study variables. Four items were used to assess each variable, and subjects responded on a 7-point scale, with 1 indicating “strongly disagree” and 7 signifying “strongly agree.”

All questions referred to the states the subjects were in immediately after the experimental manipulation. Psychological mindedness was measured using the questions: (1) New reflections about myself emerged; (2) I got to know myself better; (3) I became aware of something about myself; (4) I was in touch with my feelings. The reliability of this scale was: 0.78. Decentration was measured using the questions: (1) I looked at myself from a broader perspective; (2) I looked at myself from a distance; (3) I looked at myself from different points of view; (4) I looked at myself in a less rigid way. The reliability of this scale was: 0.76. Acceptance was measured using the questions: (1) I felt that I accepted myself as I am; (2) I felt that I accepted my different sides; (3) I was understanding of myself; (4) I was tolerant of my faults or limitations. The reliability of this scale was: 0.85. The score of each scale is the average of the four items.

#### Procedure

Participants were randomly assigned to a condition in which the multiple self was elicited or a condition in which the unitary self was elicited. We used the same technique as in the previous study. As in the previous studies, after the manipulation, subjects rated their current mood on a 1-item, 7-point scale and then answered the same questions designed to test the effectiveness of the manipulation. In the next step, participants answered 12 questions that measured psychological reflexivity, decentration, and acceptance. Finally, we checked whether participants guessed the purpose of the study and whether they were aware of the manipulation using the same questions as in the previous studies (Bargh and Chartrand, 2000).

### Results and discussion

None of the subjects guessed the purpose of the study or its experimental nature.

The experimental manipulation proved to be effective. Subjects in the multiple self-condition claimed that the task allowed them to focus on and look at different sides of their personality to a greater extent (*M* = 5.81; *SD* = 0.62) than subjects in the unitary self-condition (*M* = 3.77; *SD* = 1.04), *t*_(157)_ = 14.66; *p* < 0.001; *d* = 2.38.

As in the previous studies, mood did not change under manipulation—subjects in the multiple self-condition did not differ in mood (*M* = 4.75; *SD* = 1.3) from subjects in the unitary self-condition (*M* = 4.38; *SD* = 1.56), *t*_(157)_ = 1.61; *p* = 0.11; *d* = 0.26, ruling out mediation.

As predicted, individuals in whom the multiple self was elicited reported higher levels of psychological mindedness (*M* = 4; *SD* = 1.39) compared to individuals in whom the unitary self was elicited (*M* = 3.39; *SD* = 1.36), *t*_(157)_ = 2.78; *p* = 0.006; *d* = 0.44.

Also as predicted, subjects in whom the multiple self was induced reported higher levels of decentration (*M* = 4.28; *SD* = 1.34) compared to subjects in whom the unitary self was induced (*M* = 3.77; *SD* = 1.41), *t*_(157)_ = 2.32; *p* = 0.022; *d* = 0.37.

Contrary to our prediction, we observed no differences between these conditions in acceptance (*M* = 4.52; *SD* = 1.62 vs. *M* = 4.26; *SD* = 1.7; *t*_(157)_ = 0.99; *p* = 0.32; *d* = 0.16).

The results of the study indicates that induction of the multiple self-increases situational psychological mindedness and decentration in the subjects but has no effect on acceptance. Results show that activation of the multiple self makes people see more relationships among thoughts, feelings, and actions and more complexity in one's psyche compared to activation of the unitary self. It also helps to see oneself from a more distant perspective. However, the activation of the multiple self-did not change the tendency to make judgments or evaluations about oneself.

## General discussion

In five experiments, we demonstrated that induction of the multiple self-increased various dimensions of psychological openness compared to induction of the unitary self. We demonstrated this effect against the widest possible range of aspects of openness: openness to experience as a state (Study 1), openness to change (Study 3 and Study 4), range of values (Studies 2 and 3), psychological mindedness (Study 5), and decentering (Study 5). Some of the tools/instructions were modified to refer to states rather than enduring traits. In the conducted research we used different ways of manipulating the multiple self, which increases the reliability of the studies.

The results of this research extend our understanding of the dynamic nature of self-concept by identifying and investigating two distinct modes of self-perception that are activated in different contexts: the unitary self and the multiple self. These modes represent fundamentally different ways of experiencing and organizing self-knowledge that can be situationally induced. This research seems to be the first systematic investigation of how these distinct modes operate and influence psychological functioning.

The results also seem to confirm a mechanism that exists during psychotherapy. As we mentioned in the introduction, during psychotherapy, clients often learn to perceive or understand their experience in terms of multiple selves, i.e., they activate the multiple self-mode. In our study we tried to isolate this factor from the therapeutic context and transferred it to the laboratory. It turned out that activating the multiple self-had consequences similar to the expectations and goals adopted in psychotherapy, i.e., it increased openness to different aspects of self-experience (in other words, it decreased rigidity and inflexibility of behavior).

Our findings suggest some interesting parallels with processes observed in psychotherapy, though these parallels should be interpreted with considerable caution. As noted in the introduction, several therapeutic approaches incorporate work with different aspects of the self. In our study, we isolated one specific element—the recognition of multiple self-aspects—to examine its effects under controlled conditions. The observed increase in psychological openness aligns with some therapeutic goals related to reducing rigidity and increasing flexibility. However, it is important to note that this parallel exists only at the level of basic psychological processes.

While our research demonstrates some parallels between laboratory-induced multiple self-activation and certain aspects of psychotherapeutic processes, we acknowledge that this comparison has significant limitations. Psychotherapy, particularly approaches that engage with self-multiplicity, involves complex mechanisms that go far beyond the simple recognition of multiple aspects of the self. Our experimental conditions, especially those implemented online, cannot fully replicate the rich, multidimensional environment of therapy. Moreover, the brief nature of our interventions stands in contrast to the often long-term processes that occur in psychotherapy. It is important to clarify that our intention was not to equate our experimental manipulations with the complex processes of psychotherapy. Rather, we aimed to isolate and study one specific aspect—the recognition of multiple selves—that is often a component of various therapeutic approaches. Our findings should be interpreted as providing insight into cognitive processes related to self-concept, rather than as directly mirroring therapeutic outcomes.

These results suggest directions for future research examining whether and how multiple self-processes might operate in therapeutic contexts. Such research would need to specifically investigate how the basic mechanisms identified in our laboratory studies manifest in actual therapeutic settings, taking into account the complex, long-term nature of therapeutic change.

An additional, surprising result was that in Study 3, induction of the multiple self-increased the level of the transcending self, meaning that values related to the wellbeing and interests of others began to have greater importance. This result suggests that induction of the multiple self not only increases openness toward one's own experience but may also increase openness toward other people. In the future, it will be interesting to see if being more open toward people manifests itself in different attitudes and views about them, e.g., tolerance, less authoritarianism.

The variability in our operationalization of the multiple self-concept across studies warrants discussion. While this approach introduced some methodological complexity, we believe it ultimately strengthens our findings. The consistency of results across different activation methods suggests that the observed effects are robust and not method-dependent, potentially enhancing the generalizability of our findings.

All methods shared a common core principle: encouraging participants to recognize and reflect on multiple aspects of their self-concept. This conceptual consistency, combined with standardized manipulation checks across studies, ensured that we were activating the same underlying construct throughout our research.

We argue that using multiple methods to activate the multiple self-offers a more ecologically valid assessment of how individuals engage with their different self-aspects. Life contexts often differ in how they prompt individuals to reflect on their multifaceted self-concepts. By employing varied induction methods, we aimed to simulate this diversity and ensure that our findings are not tied to a single mode of self-reflection.

However, we acknowledge that different activation methods may potentially influence specific aspects of openness in subtle ways—a nuance our current analysis does not fully capture. Future research should consider: 1. Systematically comparing different activation methods within a single study. 2. Developing a standardized, validated measure for activating the multiple self-concept. 3. Analyzing how different activation methods might influence specific facets of openness.

The research conducted raises several open-ended questions that arose after the study was conducted. One of them is that the manifestations of openness were studied independently of each other. However, we do not exclude that they are dependent on each other. For example, psychological mindedness and decentering may be more basic processes within openness and may be de facto mediators of the influence of the induction of the multiple self on psychological openness. This issue surely requires further research.

An interesting question is whether the experimental manipulations used throughout all of our studies may have influenced the content or the structure of the self-concept. It is possible that our manipulation affected both the content and structure of the self-concept. In the first case, we simply induced the belief in participants that they either have multiple sides to their personality or, conversely, that they are unified. This belief may also be a type of theory about one's own personality (Dweck, 1999). This induced belief can be seen as a manipulation of the content of the self-concept. In the second case, through manipulation, we imposed an organization on their self-concept, thereby influencing its structure. Let's assume that the self-concept consists of elements such as adjectives. For the same individual, whose self-concept is made up of a certain number of identical adjectives, we manipulated the multiple self-condition by organizing these adjectives into separate modules, while in the unitary self-condition, we imposed that they be organized as a single set. In this way, the same content—the same adjectives—was organized in two different ways.

We are aware that the increase in psychological openness obtained in this study may have been caused by some other mechanism. The first possibility is that the observed effect can alternatively be explained by induction of the independent self-vs. the interdependent self. Whatever the case, in both theory and empirical data, there is no indication that either interdependent self-construct can affect openness. However, to rule out this alternative explanation in future research, it is worth testing whether the combined manipulation of the unitary and multiple self and the independent and interdependent self will lead to changes in openness.

The second possible alternate explanation of the obtained effect is that induction of the multiple self-caused a negative affect which in turn triggered a defense mechanism to cope with it. First, focusing on the multiplicity of self could have violated the need for consistency (Epstein, 1981). Theoretically, focusing on one's multiplicity of selves could make one's self-concept less clear or make one feel unauthentic (wearing many masks). Second, induction of the multiple self may have brought about conflicts between aspects of the self in some individuals. Indeed, other studies have shown that when active aspects of the self-come into conflict with other available aspects of the self, the latter become cognitively suppressed (Hugenberg and Bodenhausen, 2004). Thus, the increased openness of the subjects under the influence of the induction of the multiple self would be the result of a complex defense mechanism of coping with confusion and conflict. It is possible that this type of evoked emotional response is responsible for the lack of observed effect in the case of variable acceptance. It seems that while the other dimensions of openness may appear to be those that are more cognitive in nature, acceptance is more emotional in nature. Indeed, violation of the need for coherence seems to hinder acceptance.

Against this speculation is the fact that suppression could not have occurred in our study because different aspects of the self were accessible and observed simultaneously. It is also unlikely that the subjects revealed their most stressful conflicts. As for the violation of the need for coherence—it is conceivable that induction of the multiple self might entail feelings of relief or a sense of social adjustment to multiple life contexts.

Moreover, against the affect-related explanation is the fact that we did not observe changes in mood after the manipulation, a variable we controlled for. Another argument against this explanation is that in part of our research, we presented induction constructs (both conditions) in a “positive” manner during the manipulation. Adding this information led to similar results. In future research, it is worth controlling for the possibility of violating the need for consistency. To this end, for example, one might use a post-manipulation self-image clarity measurement tool such as the State Self-Concept Clarity Scale (Nezlek and Plesko, 2001).

The lack of observed effect for the acceptance variable may also be related to the fact that acceptance often takes time. It is conceivable that noticing one's different sides, including both positive and problematic sides, first causes surprise or even distress, and with time relief and acceptance. This issue requires further research, in which repeated measurements would be useful.

The third possible mechanism is that our manipulation caused a change in cognitive style, mainly the holistic vs. analytic style (Nisbett et al., 2001). Holistic thinking involves attending to wholes rather than parts, and attention to relationships of objects within a context rather than looking at objects detached from the context. Analytical style involves a focus on prominent objects in the environment and attention to the attributes of objects independent of the context. There is a chance that the unitary self-vs. multiple self-manipulation will also support analytic vs. holistic thinking, respectively. The unitary self may result from aggregating self-descriptive features across different contexts. The multiple self-manipulation however needs both holistic and analytic thinking. It is true that one can view his various selves as bound to various contexts but at the same time one can observe the whole scene as transcending various contexts. To rule out this alternative explanation it would be useful in future studies to extend the experiments with two additional conditions for thinking styles to shed light on whether priming pure analytic vs. holistic thinking (e.g., Kwan and Chiu, 2014) independent from the self-concept manipulation will affect openness.

A fourth possible mechanism involves cognitive load differences between conditions. The multiple self-condition might have imposed a higher cognitive load than the unitary self-condition, as participants had to think about and describe different aspects of themselves. This increased cognitive load could influence openness through several mechanisms. First, cognitive load may reduce reliance on automatic thinking and defensive responses (e.g., Lavie and De Fockert, 2005), potentially leading to more spontaneous and less controlled self-descriptions. Second, the mental effort required for introspection about different self-aspects might promote deeper self-reflection and greater engagement with diverse perspectives (e.g., Neys, 2006). Third, participants might interpret their increased cognitive engagement as a sign of meaningful self-exploration, affecting their responses on openness measures (e.g., Lyubomirsky et al., 2006). While our study design did not directly assess cognitive load, future research could examine this mechanism by including measures of cognitive effort and comparing the effects of multiple self-activation with other cognitively demanding tasks.

A fifth possible mechanism involves social desirability bias—participants in the multiple self-condition might have felt that showing greater openness was more socially desirable after acknowledging their different sides. After describing their different aspects, participants might have been motivated to present themselves as more flexible and open-minded to maintain consistency with their self-presentation. However, the consistency of effects across different measures, including those less susceptible to social desirability (like range of values), suggests that social desirability alone cannot fully account for our findings. Although our manipulation checks suggest that participants did not guess the study hypotheses, future research could address this alternative explanation by including social desirability scales and using more implicit measures of openness.

A sixth possible mechanism concerns self-disclosure—participants in the multiple self-condition might have felt they revealed more about themselves compared to those in the unitary self-condition, and this greater perceived self-disclosure might have led them to report being more open. However, participants in both conditions were asked to describe themselves in detail, suggesting similar levels of self-disclosure. Future research could control for this by measuring perceived self-disclosure.

Lastly one may ask whether the use of a Polish sample influenced potential cultural differences in self-multiplicity. There is no empirical data showing whether the Polish population differs from other countries in the level of self-multiplicity.

We are aware of the inadequacies of the tool used in the first study. However, we wanted to mention that personality trait questionnaires were used in other studies that employed short, one-time interventions aimed at influencing openness (Djikic et al., 2009; MacLean et al., 2011), as well as in studies using longer interventions (Hudson and Fraley, 2015; Jackson et al., 2012; Martin et al., 2014; Piedmont, 2001). These included the Big Five Inventory (John et al., 2012), NEO-FFI, and NEO PI-R (Costa and McCrae, 1992). In the future, it would be beneficial to use measures sensitive to situational influences, such as the Need for Cognitive Closure Scale (Kossowska et al., 2002). This scale represents the cognitive aspect of openness and could be more appropriate for examining how experimental manipulations affect short-term changes in openness.

It is not our argument that our manipulation caused permanent or strong personality changes in participants. Rather, we meant that the manipulation induced a temporary change in personality state, specifically, the openness state. Our study demonstrates the short-term malleability of openness in response to the activation of the multiple-self mode. However, it remains an open question whether the repeated activation of such states could lead to more enduring personality changes. Repeated engagement in the multiple-self mode may cultivate lasting openness traits by fostering habitual cognitive and emotional flexibility. This hypothesis aligns with theories of personality plasticity, which suggest that repeated activation of specific personality states can shape enduring traits over time (Fleeson, 2001; Hudson and Fraley, 2015). For example, interventions promoting mindfulness or cognitive flexibility have shown sustained increases in openness-related traits (Jackson et al., 2012; Baer, 2003). Future research could test whether repeated activations of the multiple-self mode lead to enduring personality changes, offering insights into mechanisms underlying personality plasticity and therapeutic outcomes.

We acknowledge that Study 1 lacked a direct manipulation check, which is a limitation of this research. Although manipulation checks based on the analysis of self-descriptions have been used in many priming studies (e.g., Gardner et al., 1999; Trafimow et al., 1991; Wang et al., 2011), it would be valuable to implement a more explicit manipulation check, similar to those used in Studies 2–5, in future replications or extensions of Study 1.

One limitation of the conducted research is that we focused solely on the experimental elicitation of self-constructs without considering individual differences in the multiple self. Given that people differ in terms of their multiple selves, including the complexity of the self or the clarity of the concept of self, the manipulation of unitary vs. multiple self may have different meanings for them and result in different processes. For example, for people with a more multiple structure activating the multiple self may seem more natural, whereas for people with a more unitary structure it may risk violating their image. Similarly, individuals with characterological self-image ambiguity may perceive manipulation of the multiple self-more negatively than individuals with high clarity. Although our intention was to focus on universal processes, it is not impossible that accounting for individual differences in the multiple self in future research could bring new light and perhaps lead to stronger effects.

A second limitation of our research concerns the selection of openness dimensions. While we examined six different aspects of openness (state openness, openness to values, openness to change, psychological reflexivity, decentering, and acceptance), many other important dimensions of psychological openness were not included in our research. Future research could explore whether multiple self-activation influences other dimensions of openness, providing a more comprehensive understanding of the relationship between self-organization and psychological openness.

Another limitation of the research conducted is that some of it was conducted over the Internet. Internet research carries the risk of involving several confounding variables. It is therefore worthwhile to replicate this research in a laboratory setting.

While our research focused on immediate experimental effects, the findings contribute to broader theoretical perspectives on personality development. The observed relationship between self-organization and openness suggests potential mechanisms underlying personality plasticity. Specifically, the systematic influence of multiple self-activation on various domains of openness indicates that how individuals process self-relevant information may play a crucial role in personality development. This aligns with contemporary research on personality plasticity (Roberts and Mroczek, 2008) and suggests that different modes of self-organization might serve as one pathway through which enduring personality changes occur.

## Data Availability

The raw data supporting the conclusions of this article will be made available by the authors, without undue reservation.
